# Detroit Needs Assessment Study: A Community Academic Partnership With the Detroit Area Agency on Aging

**DOI:** 10.1093/geroni/igab046.1886

**Published:** 2021-12-17

**Authors:** Faith Hopp, Fay Keys, Elizabeth Chapleski, Cheryl La Wicker, Patricia Rencher, Shirley Thomas, Anne Davis, Ronald Taylor

**Affiliations:** 1 Wayne State University, Detroit, Michigan, United States; 2 Urban Aging L. 3C, Detroit, Michigan, United States; 3 Wayne State University, Ypsilanti, Michigan, United States; 4 Detroit Area Agency on Aging, Detroit, Michigan, United States

## Abstract

This presentation discusses a comprehensive needs assessment to inform long-term strategic planning for the Detroit Area Agency on Aging. The goal was to provide in-depth input from the older population (age 60+) and key agency stakeholders, using surveys (413 community participants), listening sessions (132 participants), 23 interviews with homebound older adults, and online surveys (94) targeting medical, church, government, academic, media and HSO stakeholders. Findings indicate that many participants were not aware of available community services. For example, one-third (33.3%) had not heard of Medicaid waivers providing services outside of nursing homes, while nearly one in five (22.0%) had not heard about senior employment services. The most common areas of unmet need were for caregiver workshops (16.3%) and diabetes management classes (15.7%). Community services most often noted as ‘extremely important’ included health and wellness programs (68.8%), services to help access health and supportive services (71%), easy to find service information (67.7%), home care and housekeeping services (66.4%), and caregiver support (63.7%). Stakeholder findings provide insight regarding this lack of awareness. Asked “How familiar do you think the general public is with DAAA?” , 10.8% answered ‘very familiar’ and 33% ‘unfamiliar’. Findings related to an “Age-Friendly City” suggest the importance of access to supportive community services, transportation, safety, housing, and healthcare. Engagement of older adults in needs assessments plays a vital role in Area Agencies on Aging meeting the needs of emerging aging cohorts by developing ‘age friendly’ strategies to address increasing racial, ethnic, socioeconomic, and cultural diversity.

